# Correction: Factors in COVID-19 vaccine uptake in five racial/ethnic Colorado communities: A report from the Colorado CEAL project

**DOI:** 10.1371/journal.pone.0328097

**Published:** 2025-07-10

**Authors:** Sarah E. Brewer, Kaitlyn B. Bertin, Krithika Suresh, Crystal LoudHawk-Hedgepeth, Montelle Tamez, Jenna E. Reno, Bethany M. Kwan, Donald E. Nease

There are errors in the Funding statement. The correct Funding statement is as follows: Research reported in this publication was supported by the National Institutes of Health (NIH) under the Sub-OTA No. 6793-02-S018. This research was, in part, funded by the NIH Agreement OT2HL158287. The views and conclusions contained in this document are those of the authors and should not be interpreted as representing the official policies, either expressed or implied, of the NIH. The content is solely the responsibility of the authors and does not necessarily represent the official views of the National Institutes of Health. This project and related publication were supported by NIH/NCATS Colorado CTSA Grant Number UL1 TR002535. Its contents are the authors’ sole responsibility and do not necessarily represent official NIH views. The funders had no role in study design, data collection and analysis, decision to publish, or preparation of the manuscript. Study data were collected and managed using REDCap electronic data capture tools hosted at the University of Colorado. REDCap (Research Electronic Data Capture) is a secure, web-based application designed to support data capture for research studies, providing: 1) an intuitive interface for validated data entry; 2) audit trails for tracking data manipulation and export procedures; 3) automated export procedures for seamless data downloads to common statistical packages; and 4) procedures for importing data from external sources.

## References

[1] Brewer SE, Bertin KB, Suresh K, LoudHawk-Hedgepeth C, Tamez M, Reno JE, et al. Factors in COVID-19 vaccine uptake in five racial/ethnic Colorado communities: A report from the Colorado CEAL project. PLoS One. 2024;19(6):e0305160. doi: 10.1371/journal.pone.0305160 38865424 PMC11168616

