# Supplementary material for: Factors in COVID-19 vaccine uptake in five racial/ethnic Colorado communities: A report from the Colorado CEAL project
Source: PLoS One. 2024 Jun 12;19(6):e0305160. doi: 10.1371/journal.pone.0305160 (PMC11168616; doi:10.1371/journal.pone.0305160)
Supplement: S1 File — S1a-S1f Table. (PDF) [file pone.0305160.s001.pdf]

**Supplemental Table 1a:** Motivators (reasons) for getting a COVID-19 vaccine, overall and by community cohort

| Motivator | CO-CEAL Sample Overall (N = 812) | | Urban Latino/a/x (N = 189) | | Rural Latino/a/x (N = 195) | | Urban Black/AA (N = 169) | | Rural Black/AA (N = 195) | | Urban AI/AN (N = 64) | |
|---|---|---|---|---|---|---|---|---|---|---|---|---|
| | **Rank** | **n (%)** | **Rank** | **n (%)** | **Rank** | **n (%)** | **Rank** | **n (%)** | **Rank** | **n (%)** | **Rank** | **n (%)** |
| I want to keep my family safe | 1 | 567 (69.8%) | 1 | 131 (69.3%) | 1 | 149 (76.4%) | 1 | 96 (56.8%) | 1 | 141 (72.3%) | 1 | 50 (78.1%) |
| I want to keep myself safe | 2 | 472 (58.1%) | 2 | 110 (58.2%) | 2 | 112 (57.4%) | 2 | 75 (44.4%) | 2 | 131 (67.2%) | 2 | 44 (68.8%) |
| I want to keep my community safe | 3 | 411 (50.6%) | 3 | 97 (51.3%) | 3 | 90 (46.2%) | 3 | 58 (34.3%) | 3 | 130 (66.7%) | 3 | 36 (56.3%) |
| I want to feel safe around other people | 4 | 285 (35.1%) | 5 | 89 (47.1%) | 4 | 77 (39.5%) | 5 | 42 (24.9%) | 4 | 48 (24.6%) | 4 | 29 (45.3%) |
| I don't want to get really sick from COVID-19 | 5 | 265 (32.6%) | 4 | 92 (48.7%) | 5 | 73 (37.4%) | 4 | 45 (26.6%) | 5 | 26 (13.3%) | 4 | 29 (45.3%) |
| I believe life won't go back to normal until most people get a COVID-19 vaccine | 6 | 198 (24.4%) | 6 | 75 (39.7%) | 7 | 50 (25.6%) | 7 | 29 (17.2%) | 6 | 25 (12.8%) | 7 | 19 (29.7%) |
| I want to stop wearing masks | 7 | 159 (19.6%) | 7 | 56 (29.6%) | 6 | 57 (29.2%) | 9 | 20 (11.8%) | 9 | 10 (5.1%) | 8 | 16 (25.0%) |
| It is a requirement for my school or workplace | 8 | 115 (14.2%) | 9 | 35 (18.5%) | 8 | 28 (14.4%) | 11 | 19 (11.2%) | 11 | 5 (2.6%) | 6 | 28 (43.8%) |
| I have a chronic health problem, like asthma or diabetes | 9 | 103 (12.7%) | 8 | 37 (19.6%) | 9 | 27 (13.8%) | 9 | 20 (11.8%) | 10 | 8 (4.1%) | 9 | 11 (17.2%) |
| My doctor told me to get a COVID-19 vaccine | 10 | 88 (10.8%) | 10 | 27 (14.3%) | 10 | 19 (9.7%) | 8 | 22 (13.0%) | 7 | 14 (7.2%) | 10 | 6 (9.4%) |
| None | 11 | 67 (8.3%) | 11 | 15 (7.9%) | 12 | 9 (4.6%) | 6 | 30 (17.8%) | 8 | 13 (6.7%) | | |
| Other (unspecified) | 12 | 46 (5.7%) | 12 | 8 (4.2%) | 11 | 12 (6.2%) | 12 | 15 (8.9%) | 11 | 5 (2.6%) | 10 | 6 (9.4%) |
| Did not respond (missing) | | 19 (2.3%) | | 4 (2.1%) | | 11 (5.6%) | | 3 (1.8%) | | | | 1 (1.6%) |
| Selected any response (including 'Other' or 'None') | | 793 (97.7%) | | 185 (97.9%) | | 184 (94.4%) | | 166 (98.2%) | | 195 (100%) | | 63 (98.4%) |
| Selected any response (besides 'None') | | 726 (89.4%) | | 170 (89.9%) | | 175 (89.7%) | | 136 (80.5%) | | 182 (93.3%) | | 63 (98.4%) |
| Selected any response (besides 'Other' or 'None') | | 692 (85.2%) | | 163 (86.2%) | | 166 (85.1%) | | 125 (74.0%) | | 178 (91.3%) | | 60 (93.8%) |

**Supplemental Table 1b:** Motivators (reasons) for getting a COVID-19 vaccine among those who had initiated, overall and by community cohort

| | Initiated COVID-19 vaccine | | | | | | | | | | | |
|---|---|---|---|---|---|---|---|---|---|---|---|---|
| | **CO-CEAL Sample Overall (N = 577)** | | **Urban Latino/a/x (N = 140)** | | **Rural Latino/a/x (N = 144)** | | **Urban Black/AA (N = 105)** | | **Rural Black/AA (N = 128)** | | **Urban AI/AN (N = 60)** | |
| **Motivator** | **Rank** | **n (%)** | **Rank** | **n (%)** | **Rank** | **n (%)** | **Rank** | **n (%)** | **Rank** | **n (%)** | **Rank** | **n (%)** |
| I want to keep my family safe | 1 | 471 (81.6%) | 1 | 113 (80.7%) | 1 | 129 (89.6%) | 1 | 81 (77.1%) | 1 | 98 (76.6%) | 1 | 50 (83.3%) |
| I want to keep myself safe | 2 | 402 (69.7%) | 2 | 96 (68.6%) | 2 | 100 (69.4%) | 2 | 65 (61.9%) | 1 | 98 (76.6%) | 2 | 43 (71.7%) |
| I want to keep my community safe | 3 | 339 (58.8%) | 3 | 85 (60.7%) | 3 | 80 (55.6%) | 3 | 51 (48.6%) | 3 | 87 (68.0%) | 3 | 36 (60.0%) |
| I want to feel safe around other people | 4 | 248 (43.0%) | 4 | 80 (57.1%) | 4 | 70 (48.6%) | 5 | 36 (34.3%) | 4 | 33 (25.8%) | 4 | 29 (48.3%) |
| I don't want to get really sick from COVID-19 | 5 | 227 (39.3%) | 4 | 80 (57.1%) | 5 | 64 (44.4%) | 4 | 37 (35.2%) | 5 | 18 (14.1%) | 5 | 28 (46.7%) |
| I believe life won't go back to normal until most people get a COVID-19 vaccine | 6 | 174 (30.2%) | 6 | 70 (50.0%) | 7 | 44 (30.6%) | 6 | 25 (23.8%) | 6 | 16 (12.5%) | 7 | 19 (31.7%) |
| I want to stop wearing masks | 7 | 139 (24.1%) | 7 | 49 (35.0%) | 6 | 48 (33.3%) | 7 | 19 (18.1%) | 8 | 7 (5.5%) | 8 | 16 (26.7%) |
| It is a requirement for my school or workplace | 8 | 106 (18.4%) | 9 | 31 (22.1%) | 8 | 26 (18.1%) | 9 | 16 (15.2%) | 9 | 5 (3.9%) | 5 | 28 (46.7%) |
| I have a chronic health problem, like asthma or diabetes | 9 | 89 (15.4%) | 8 | 32 (22.9%) | 8 | 26 (18.1%) | 9 | 16 (15.2%) | 9 | 5 (3.9%) | 9 | 10 (16.7%) |
| My doctor told me to get a COVID-19 vaccine | 10 | 80 (13.9%) | 10 | 25 (17.9%) | 10 | 19 (13.2%) | 8 | 17 (16.2%) | 7 | 13 (10.2%) | 10 | 6 (10.0%) |
| Other (unspecified) | 11 | 19 (3.3%) | 11 | 1 (0.7%) | 11 | 4 (2.8%) | 11 | 8 (7.6%) | 11 | 2 (1.6%) | 11 | 4 (6.7%) |
| Did not respond (missing) | | 5 (0.9%) | | 1 (0.7%) | | 2 (1.4%) | | 1 (1.0%) | | | | 1 (1.7%) |
| Selected any response (including 'Other') | | 572 (99.1%) | | 139 (99.3%) | | 142 (98.6%) | | 104 (99.0%) | | 128 (100%) | | 59 (98.3%) |
| Selected any response (besides 'Other') | | 561 (97.2%) | | 138 (98.6%) | | 141 (97.9%) | | 99 (94.3%) | | 126 (98.4%) | | 57 (95.0%) |

**Supplemental Table 1c:** Motivators (reasons) for getting a COVID-19 vaccine among those who had not initiated, overall and by community cohort

| | Did not initiate COVID-19 vaccine | | | | | | | | | | | |
|---|---|---|---|---|---|---|---|---|---|---|---|---|
| | CO-CEAL Sample Overall (N = 235) | | Urban Latino/a/x (N = 49) | | Rural Latino/a/x x (N = 51) | | Urban Black/AA (N = 64) | | Rural Black/AA (N = 67) | | Urban AI/AN (N = 4) | |
| **Motivator** | **Rank** | **n (%)** | **Rank** | **n (%)** | **Rank** | **n (%)** | **Rank** | **n (%)** | **Rank** | **n (%)** | **Rank** | **n (%)** |
| I want to keep my family safe | 1 | 96 (40.9%) | 1 | 18 (36.7%) | 1 | 20 (39.2%) | 2 | 15 (23.4%) | 1 | 43 (64.2%) | | |
| I want to keep my community safe | 2 | 72 (30.6%) | 4 | 12 (24.5%) | 3 | 10 (19.6%) | 5 | 7 (10.9%) | 1 | 43 (64.2%) | | |
| I want to keep myself safe | 3 | 70 (29.8%) | 3 | 14 (28.6%) | 2 | 12 (23.5%) | 3 | 10 (15.6%) | 3 | 33 (49.3%) | 2 | 1 (25.0%) |
| None | 4 | 67 (28.5%) | 2 | 15 (30.6%) | 4 | 9 (17.6%) | 1 | 30 (46.9%) | 5 | 13 (19.4%) | | |
| I don't want to get really sick from COVID-19 | 5 | 38 (16.2%) | 4 | 12 (24.5%) | 4 | 9 (17.6%) | 4 | 8 (12.5%) | 7 | 8 (11.9%) | 2 | 1 (25.0%) |
| I want to feel safe around other people | 6 | 37 (15.7%) | 6 | 9 (18.4%) | 8 | 7 (13.7%) | 7 | 6 (9.4%) | 4 | 15 (22.4%) | | |
| Other (unspecified) | 7 | 27 (11.5%) | 7 | 7 (14.3%) | 7 | 8 (15.7%) | 5 | 7 (10.9%) | 8 | 3 (4.5%) | 1 | 2 (50.0%) |
| I believe life won't go back to normal until most people get a COVID-19 vaccine | 8 | 24 (10.2%) | 9 | 5 (10.2%) | 9 | 6 (11.8%) | 9 | 4 (6.3%) | 6 | 9 (13.4%) | | |
| I want to stop wearing masks | 9 | 20 (8.5%) | 7 | 7 (14.3%) | 4 | 9 (17.6%) | 12 | 1 (1.6%) | 8 | 3 (4.5%) | | |
| I have a chronic health problem, like asthma or diabetes | 10 | 14 (6.0%) | 9 | 5 (10.2%) | 11 | 1 (2.0%) | 9 | 4 (6.3%) | 8 | 3 (4.5%) | 2 | 1 (25.0%) |
| It is a requirement for my school or workplace | 11 | 9 (3.8%) | 11 | 4 (8.2%) | 10 | 2 (3.9%) | 11 | 3 (4.7%) | | | | |
| My doctor told me to get a COVID-19 vaccine | 12 | 8 (3.4%) | 12 | 2 (4.1%) | | | 8 | 5 (7.8%) | 11 | 1 (1.5%) | | |
| Did not respond (missing) | | 14 (6.0%) | | 3 (6.1%) | | 9 (17.6%) | | 2 (3.1%) | | | | |
| Selected any response (including 'Other' or 'None') | | 221 (94.0%) | | 46 (93.9%) | | 42 (82.4%) | | 62 (96.9%) | | 67 (100%) | | 4 (100%) |
| Selected any response (besides 'None') | | 154 (65.5%) | | 31 (63.3%) | | 33 (64.7%) | | 32 (50.0%) | | 54 (80.6%) | | 4 (100%) |
| Selected any response (besides 'Other' or 'None') | | 131 (55.7%) | | 25 (51.0%) | | 25 (49.0%) | | 26 (40.6%) | | 52 (77.6%) | | 3 (75.0%) |

**Supplemental Table 1d:** Concerns about (reasons against) getting a COVID-19 vaccine, overall and by community cohort

| Concern | CO-CEAL Sample Overall (N = 812) | | Urban Latino/a/x (N = 189) | | Rural Latino/a/x (N = 195) | | Urban Black/AA (N = 169) | | Rural Black/AA (N = 195) | | Urban AI/AN (N = 64) | |
|---|---|---|---|---|---|---|---|---|---|---|---|---|
| | Rank | n (%) | Rank | n (%) | Rank | n (%) | Rank | n (%) | Rank | n (%) | Rank | n (%) |
| I'm concerned about side effects from the vaccine | 1 | 258 (31.8%) | 2 | 51 (27.0%) | 1 | 64 (32.8%) | 1 | 60 (35.5%) | 1 | 68 (34.9%) | 2 | 15 (23.4%) |
| None | 2 | 178 (21.9%) | 1 | 68 (36.0%) | 5 | 24 (12.3%) | 6 | 28 (16.6%) | 2 | 31 (15.9%) | 1 | 27 (42.2%) |
| I don't trust that the vaccine will be safe | 3 | 125 (15.4%) | 3 | 32 (16.9%) | 3 | 37 (19.0%) | 2 | 37 (21.9%) | 8 | 14 (7.2%) | 7 | 5 (7.8%) |
| I don't know enough about how well a COVID-19 vaccine works | 4 | 119 (14.7%) | 4 | 24 (12.7%) | 2 | 42 (21.5%) | 3 | 31 (18.3%) | 6 | 16 (8.2%) | 4 | 6 (9.4%) |
| I'm not concerned about getting really sick from COVID-19 | 5 | 98 (12.1%) | 7 | 15 (7.9%) | 6 | 22 (11.3%) | 4 | 29 (17.2%) | 3 | 27 (13.8%) | 7 | 5 (7.8%) |
| I don't like needles | 6 | 95 (11.7%) | 5 | 17 (9.0%) | 7 | 21 (10.8%) | 4 | 29 (17.2%) | 4 | 21 (10.8%) | 3 | 7 (10.9%) |
| Other (unspecified) | 7 | 71 (8.7%) | 8 | 14 (7.4%) | 4 | 27 (13.8%) | 18 | 9 (5.3%) | 5 | 19 (9.7%) | 9 | 2 (3.1%) |
| I don't think vaccines work very well | 8 | 61 (7.5%) | 9 | 12 (6.3%) | 10 | 13 (6.7%) | 11 | 20 (11.8%) | 7 | 15 (7.7%) | 14 | 1 (1.6%) |
| I am concerned COVID-19 vaccines may cause infertility | 9 | 53 (6.5%) | 9 | 12 (6.3%) | 9 | 14 (7.2%) | 14 | 14 (8.3%) | 9 | 7 (3.6%) | 4 | 6 (9.4%) |
| I am concerned the COVID-19 vaccines contain fetal cells | 10 | 50 (6.2%) | 9 | 12 (6.3%) | 11 | 12 (6.2%) | 10 | 21 (12.4%) | 17 | 3 (1.5%) | 9 | 2 (3.1%) |
| I already had COVID-19 | 11 | 48 (5.9%) | 5 | 17 (9.0%) | 8 | 15 (7.7%) | 14 | 14 (8.3%) | 19 | 2 (1.0%) | | |
| I have religious reasons not to vaccinate | 12 | 47 (5.8%) | 12 | 11 (5.8%) | 12 | 11 (5.6%) | 8 | 23 (13.6%) | 21 | 1 (0.5%) | 14 | 1 (1.6%) |
| I am concerned I could get COVID-19 from the vaccine | 13 | 43 (5.3%) | 14 | 10 (5.3%) | 17 | 6 (3.1%) | 9 | 22 (13.0%) | 17 | 3 (1.5%) | 9 | 2 (3.1%) |
| I am concerned I may need to miss work if I feel sick from the COVID-19 vaccine | 14 | 42 (5.2%) | 12 | 11 (5.8%) | 15 | 9 (4.6%) | 17 | 11 (6.5%) | 11 | 5 (2.6%) | 4 | 6 (9.4%) |
| I don't believe the COVID-19 pandemic is as bad as some people say it is | 15 | 39 (4.8%) | 19 | 5 (2.6%) | 13 | 10 (5.1%) | 12 | 17 (10.1%) | 10 | 6 (3.1%) | 14 | 1 (1.6%) |
| I am concerned COVID-19 vaccines could change your DNA | 15 | 39 (4.8%) | 15 | 8 (4.2%) | 18 | 5 (2.6%) | 7 | 24 (14.2%) | | | 9 | 2 (3.1%) |

| Concern | CO-CEAL Sample Overall (N = 812) | | Urban Latino/a/x (N = 189) | | Rural Latino/a/x (N = 195) | | Urban Black/AA (N = 169) | | Rural Black/AA (N = 195) | | Urban AI/AN (N = 64) | |
|---|---|---|---|---|---|---|---|---|---|---|---|---|
| | **Rank** | **n (%)** | **Rank** | **n (%)** | **Rank** | **n (%)** | **Rank** | **n (%)** | **Rank** | **n (%)** | **Rank** | **n (%)** |
| I'm allergic to vaccines | 17 | 36 (4.4%) | 17 | 7 (3.7%) | 13 | 10 (5.1%) | 13 | 15 (8.9%) | 13 | 4 (2.1%) | | |
| I don't want to pay for it | 18 | 32 (3.9%) | 18 | 6 (3.2%) | 16 | 7 (3.6%) | 16 | 13 (7.7%) | 13 | 4 (2.1%) | 9 | 2 (3.1%) |
| I don't know when or where to get a COVID-19 vaccine | 19 | 21 (2.6%) | 15 | 8 (4.2%) | 19 | 3 (1.5%) | 22 | 5 (3.0%) | 11 | 5 (2.6%) | | |
| I am concerned I will need to provide a social security number or government ID to get the COVID-19 vaccine | 20 | 19 (2.3%) | 19 | 5 (2.6%) | 22 | 1 (0.5%) | 18 | 9 (5.3%) | 13 | 4 (2.1%) | | |
| I cannot get the vaccine from a place that I trust | 21 | 18 (2.2%) | 21 | 2 (1.1%) | 19 | 3 (1.5%) | 18 | 9 (5.3%) | 13 | 4 (2.1%) | | |
| It is difficult to get to a vaccination site to get the COVID-19 vaccine | 22 | 15 (1.8%) | 21 | 2 (1.1%) | 19 | 3 (1.5%) | 21 | 8 (4.7%) | 19 | 2 (1.0%) | | |
| Did not respond (missing) | | 65 (8.0%) | | 13 (6.9%) | | 38 (19.5%) | | 9 (5.3%) | | 1 (0.5%) | | 4 (6.3%) |
| Selected any response (including ‘Other’ or ‘None’) | | 747 (92.0%) | | 176 (93.1%) | | 157 (80.5%) | | 160 (94.7%) | | 194 (99.5%) | | 60 (93.8%) |
| Selected any response (besides ‘None’) | | 569 (70.1%) | | 108 (57.1%) | | 133 (68.2%) | | 132 (78.1%) | | 163 (83.6%) | | 33 (51.6%) |
| Selected any response (besides ‘Other’ or ‘None’) | | 689 (84.9%) | | 164 (86.8%) | | 138 (70.8%) | | 153 (90.5%) | | 175 (89.7%) | | 59 (92.2%) |

**Supplemental Table 1e:** Concerns about (reasons against) getting a COVID-19 vaccine among those who had not initiated, overall and by community cohort

| | Did not initiate COVID-19 vaccine | | | | | | | | | | | |
|---|---|---|---|---|---|---|---|---|---|---|---|---|
| | CO-CEAL Sample Overall (N = 235) | | Urban Latino/a/x (N = 49) | | Rural Latino/a/x (N = 51) | | Urban Black/AA (N = 64) | | Rural Black/AA (N = 67) | | Urban AI/AN (N = 4) | |
| **Concern** | **Rank** | **n (%)** | **Rank** | **n (%)** | **Rank** | **n (%)** | **Rank** | **n (%)** | **Rank** | **n (%)** | **Rank** | **n (%)** |
| I'm concerned about side effects from the vaccine | 1 | 90 (38.3%) | 1 | 20 (40.8%) | 1 | 21 (41.2%) | 1 | 24 (37.5%) | 1 | 23 (34.3%) | 1 | 2 (50.0%) |
| I don't trust that the vaccine will be safe | 2 | 64 (27.2%) | 1 | 20 (40.8%) | 2 | 19 (37.3%) | 2 | 21 (32.8%) | 8 | 3 (4.5%) | 2 | 1 (25.0%) |
| Other (unspecified) | 3 | 44 (18.7%) | 3 | 9 (18.4%) | 3 | 12 (23.5%) | 21 | 3 (4.7%) | 2 | 19 (28.4%) | 2 | 1 (25.0%) |
| I'm not concerned about getting really sick from COVID-19 | 4 | 42 (17.9%) | 5 | 8 (16.3%) | 5 | 9 (17.6%) | 5 | 17 (26.6%) | 4 | 7 (10.4%) | 2 | 1 (25.0%) |
| I don't know enough about how well a COVID-19 vaccine works | 4 | 42 (17.9%) | 3 | 9 (18.4%) | 4 | 11 (21.6%) | 8 | 16 (25.0%) | 5 | 6 (9.0%) | | |
| I don't like needles | 6 | 39 (16.6%) | 7 | 7 (14.3%) | 7 | 7 (13.7%) | 9 | 14 (21.9%) | 3 | 11 (16.4%) | | |
| I am concerned the COVID-19 vaccines contain fetal cells | 7 | 33 (14.0%) | 7 | 7 (14.3%) | 7 | 7 (13.7%) | 4 | 19 (29.7%) | | | | |
| I have religious reasons not to vaccinate | 7 | 33 (14.0%) | 7 | 7 (14.3%) | 6 | 8 (15.7%) | 5 | 17 (26.6%) | | | 2 | 1 (25.0%) |
| I don't think vaccines work very well | 9 | 27 (11.5%) | 14 | 3 (6.1%) | 9 | 6 (11.8%) | 9 | 14 (21.9%) | 6 | 4 (6.0%) | | |
| I am concerned COVID-19 vaccines may cause infertility | 10 | 26 (11.1%) | 7 | 7 (14.3%) | 12 | 4 (7.8%) | 11 | 12 (18.8%) | 9 | 2 (3.0%) | 2 | 1 (25.0%) |
| I am concerned COVID-19 vaccines could change your DNA | 10 | 26 (11.1%) | 13 | 4 (8.2%) | 13 | 2 (3.9%) | 3 | 20 (31.3%) | | | | |
| I am concerned I could get COVID-19 from the vaccine | 12 | 24 (10.2%) | 11 | 5 (10.2%) | | | 5 | 17 (26.6%) | 9 | 2 (3.0%) | | |
| I already had COVID-19 | 13 | 22 (9.4%) | 5 | 8 (16.3%) | 10 | 5 (9.8%) | 13 | 8 (12.5%) | 12 | 1 (1.5%) | | |
| I don't believe the COVID-19 pandemic is as bad as some people say it is | 14 | 21 (8.9%) | 14 | 3 (6.1%) | 10 | 5 (9.8%) | 11 | 12 (18.8%) | 12 | 1 (1.5%) | | |

| Concern | Did not initiate COVID-19 vaccine | | | | | | | | | | | |
|---|---|---|---|---|---|---|---|---|---|---|---|---|
| | CO-CEAL Sample Overall (N = 235) | | Urban Latino/a/x (N = 49) | | Rural Latino/a/x (N = 51) | | Urban Black/AA (N = 64) | | Rural Black/AA (N = 67) | | Urban AI/AN (N = 4) | |
| | Rank | n (%) | Rank | n (%) | Rank | n (%) | Rank | n (%) | Rank | n (%) | Rank | n (%) |
| I am concerned I may need to miss work if I feel sick from the COVID-19 vaccine | 15 | 17 (7.2%) | 11 | 5 (10.2%) | 13 | 2 (3.9%) | 13 | 8 (12.5%) | 12 | 1 (1.5%) | 2 | 1 (25.0%) |
| I'm allergic to vaccines | 16 | 13 (5.5%) | 18 | 1 (2.0%) | 15 | 1 (2.0%) | 17 | 7 (10.9%) | 6 | 4 (6.0%) | | |
| I don't want to pay for it | 17 | 10 (4.3%) | 16 | 2 (4.1%) | | | 13 | 8 (12.5%) | | | | |
| I cannot get the vaccine from a place that I trust | 17 | 10 (4.3%) | 18 | 1 (2.0%) | 15 | 1 (2.0%) | 17 | 7 (10.9%) | 12 | 1 (1.5%) | | |
| I am concerned I will need to provide a social security number or government ID to get the COVID-19 vaccine | 17 | 10 (4.3%) | | | 15 | 1 (2.0%) | 13 | 8 (12.5%) | 12 | 1 (1.5%) | | |
| I don't know when or where to get a COVID-19 vaccine | 20 | 9 (3.8%) | 16 | 2 (4.1%) | | | 20 | 5 (7.8%) | 9 | 2 (3.0%) | | |
| It is difficult to get to a vaccination site to get the COVID-19 vaccine | 21 | 8 (3.4%) | | | | | 17 | 7 (10.9%) | 12 | 1 (1.5%) | | |
| Did not respond (missing) | | 12 (5.1%) | | 3 (6.1%) | | 5 (9.8%) | | 3 (4.7%) | | 1 (1.5%) | | |
| Selected any response (including 'Other') | | 223 (94.9%) | | 46 (93.9%) | | 46 (90.2%) | | 61 (95.3%) | | 66 (98.5%) | | 4 (100%) |
| Selected any response (besides 'Other') | | 184 (78.3%) | | 38 (77.6%) | | 38 (74.5%) | | 58 (90.6%) | | 47 (70.1%) | | 3 (75.0%) |

**Supplemental Table 1f:** Concerns about (reasons against) getting a COVID-19 vaccine among those who had initiated, overall and by community cohort

| | Initiated COVID-19 vaccine | | | | | | | | | | | |
|---|---|---|---|---|---|---|---|---|---|---|---|---|
| | CO-CEAL Sample Overall (N = 577) | | Urban Latino/a/x (N = 140) | | Rural Latino/a/x (N = 144) | | Urban Black/AA (N = 105) | | Rural Black/AA (N = 128) | | Urban AI/AN (N = 60) | |
| **Concern** | **Rank** | **n (%)** | **Rank** | **n (%)** | **Rank** | **n (%)** | **Rank** | **n (%)** | **Rank** | **n (%)** | **Rank** | **n (%)** |
| None | 1 | 178 (30.8%) | 1 | 68 (48.6%) | 3 | 24 (16.7%) | 2 | 28 (26.7%) | 2 | 31 (24.2%) | 1 | 27 (45.0%) |
| I'm concerned about side effects from the vaccine | 2 | 168 (29.1%) | 2 | 31 (22.1%) | 1 | 43 (29.9%) | 1 | 36 (34.3%) | 1 | 45 (35.2%) | 2 | 13 (21.7%) |
| I don't know enough about how well a COVID-19 vaccine works | 3 | 77 (13.3%) | 3 | 15 (10.7%) | 2 | 31 (21.5%) | 4 | 15 (14.3%) | 6 | 10 (7.8%) | 4 | 6 (10.0%) |
| I don't trust that the vaccine will be safe | 4 | 61 (10.6%) | 4 | 12 (8.6%) | 4 | 18 (12.5%) | 3 | 16 (15.2%) | 4 | 11 (8.6%) | 7 | 4 (6.7%) |
| I don't like needles | 5 | 56 (9.7%) | 5 | 10 (7.1%) | 6 | 14 (9.7%) | 4 | 15 (14.3%) | 6 | 10 (7.8%) | 3 | 7 (11.7%) |
| I'm not concerned about getting really sick from COVID-19 | 5 | 56 (9.7%) | 8 | 7 (5.0%) | 7 | 13 (9.0%) | 6 | 12 (11.4%) | 3 | 20 (15.6%) | 7 | 4 (6.7%) |
| I don't think vaccines work very well | 7 | 34 (5.9%) | 6 | 9 (6.4%) | 11 | 7 (4.9%) | 8 | 6 (5.7%) | 4 | 11 (8.6%) | 13 | 1 (1.7%) |
| I am concerned COVID-19 vaccines may cause infertility | 8 | 27 (4.7%) | 12 | 5 (3.6%) | 8 | 10 (6.9%) | 17 | 2 (1.9%) | 8 | 5 (3.9%) | 5 | 5 (8.3%) |
| Other (unspecified) | 8 | 27 (4.7%) | 12 | 5 (3.6%) | 5 | 15 (10.4%) | 8 | 6 (5.7%) | | | 13 | 1 (1.7%) |
| I already had COVID-19 | 10 | 26 (4.5%) | 6 | 9 (6.4%) | 8 | 10 (6.9%) | 8 | 6 (5.7%) | 16 | 1 (0.8%) | | |
| I am concerned I may need to miss work if I feel sick from the COVID-19 vaccine | 11 | 25 (4.3%) | 9 | 6 (4.3%) | 11 | 7 (4.9%) | 16 | 3 (2.9%) | 10 | 4 (3.1%) | 5 | 5 (8.3%) |
| I'm allergic to vaccines | 12 | 23 (4.0%) | 9 | 6 (4.3%) | 10 | 9 (6.3%) | 7 | 8 (7.6%) | | | | |
| I don't want to pay for it | 13 | 22 (3.8%) | 17 | 4 (2.9%) | 11 | 7 (4.9%) | 12 | 5 (4.8%) | 10 | 4 (3.1%) | 9 | 2 (3.3%) |
| I am concerned I could get COVID-19 from the vaccine | 14 | 19 (3.3%) | 12 | 5 (3.6%) | 14 | 6 (4.2%) | 12 | 5 (4.8%) | 16 | 1 (0.8%) | 9 | 2 (3.3%) |
| I don't believe the COVID-19 pandemic is as bad as some people say it is | 15 | 18 (3.1%) | 20 | 2 (1.4%) | 15 | 5 (3.5%) | 12 | 5 (4.8%) | 8 | 5 (3.9%) | 13 | 1 (1.7%) |
| I am concerned the COVID-19 vaccines contain fetal cells | 16 | 17 (2.9%) | 12 | 5 (3.6%) | 15 | 5 (3.5%) | 17 | 2 (1.9%) | 12 | 3 (2.3%) | 9 | 2 (3.3%) |

| | Initiated COVID-19 vaccine | | | | | | | | | | | |
|---|---|---|---|---|---|---|---|---|---|---|---|---|
| | CO-CEAL Sample Overall (N = 577) | | Urban Latino/a/x (N = 140) | | Rural Latino/a/x (N = 144) | | Urban Black/AA (N = 105) | | Rural Black/AA (N = 128) | | Urban AI/AN (N = 60) | |
| **Concern** | **Rank** | **n (%)** | **Rank** | **n (%)** | **Rank** | **n (%)** | **Rank** | **n (%)** | **Rank** | **n (%)** | **Rank** | **n (%)** |
| I have religious reasons not to vaccinate | 17 | 14 (2.4%) | 17 | 4 (2.9%) | 17 | 3 (2.1%) | 8 | 6 (5.7%) | 16 | 1 (0.8%) | | |
| I am concerned COVID-19 vaccines could change your DNA | 18 | 13 (2.3%) | 17 | 4 (2.9%) | 17 | 3 (2.1%) | 15 | 4 (3.8%) | | | 9 | 2 (3.3%) |
| I don't know when or where to get a COVID-19 vaccine | 19 | 12 (2.1%) | 9 | 6 (4.3%) | 17 | 3 (2.1%) | | | 12 | 3 (2.3%) | | |
| Selected any response (including 'Other' or 'None') | | 524 (90.8%) | | 130 (92.9%) | | 111 (77.1%) | | 99 (94.3%) | | 128 (100%) | | 56 (93.3%) |
| Selected any response (besides 'None') | | 346 (60.0%) | | 62 (44.3%) | | 87 (60.4%) | | 71 (67.6%) | | 97 (75.8%) | | 29 (48.3%) |
| Selected any response (besides 'Other' or 'None') | | 505 (87.5%) | | 126 (90.0%) | | 100 (69.4%) | | 95 (90.5%) | | 128 (100%) | | 56 (93.3%) |
