# Supplementary material for: Factors in COVID-19 vaccine uptake in five racial/ethnic Colorado communities: A report from the Colorado CEAL project
Source: PLoS One. 2024 Jun 12;19(6):e0305160. doi: 10.1371/journal.pone.0305160 (PMC11168616; doi:10.1371/journal.pone.0305160)
Supplement: Attachment — Submitted filename: Review to manuscript number PONE-D-24-00188 PLOS ONE.pdf [file pone.0305160.s002.pdf]

Dear Professor WajdyJum'ah Al-Awaida; the academic editor of PLOS ONE:

My name is Sima Zein, PhD, I was kindly invited from you to review the manuscript entitled; Factors in COVID-19 Vaccine Uptake in Five Racial/Ethnic Colorado Communities: A report from the Colorado CEAL Project; PONE-D-24-00188.

My comments are as the following:

1) The manuscript is well written.

2) The results and methodology are convincing.

3) The only point that is concerning about the manuscript is the small sample size that were studied; 841 participants were involved in the study but 29 participants were excluded from the study, making the total participants 812. Hence, although the results are convincing, but I don't believe that the 812 participants are enough to generalize the results that authors are presenting.

4) Hence my recommendation, is to involve more participants (if possible), this would enhance the strength of the manuscript and its conclusions.

Wishing the authors the best of luck.

Sincerely,

Sima Zein, PhD

Associate Professor

Molecular and Cell Biology

S
