# Supplementary material for: Factors in COVID-19 vaccine uptake in five racial/ethnic Colorado communities: A report from the Colorado CEAL project
Source: PLoS One. 2024 Jun 12;19(6):e0305160. doi: 10.1371/journal.pone.0305160 (PMC11168616; doi:10.1371/journal.pone.0305160)
Supplement: Attachment — Submitted filename: review of the manuscript- PONE-D-24-00188.docx [file pone.0305160.s003.docx]

Based on the detailed review of the manuscript "Factors in COVID-19 Vaccine Uptake in Five Racial/Ethnic Colorado Communities: A report from the Colorado CEAL Project," here are the comments, limitations, and suggestions for improvement. The manuscript provides valuable insights into vaccine uptake within diverse communities in Colorado, focusing on various motivators and barriers to COVID-19 vaccination. It employs a robust community-based data collection methodology, ensuring relevant and contextual data gathering from targeted communities.

**Abstract:** The abstract concisely outlines the study's purpose, methodology, results, and implications. It successfully highlights the importance of tailored community messaging to improve vaccination rates. However, it could benefit from mentioning the study's limitations to provide a more balanced overview.

**Introduction:** The introduction effectively sets the stage for the study by discussing the COVID-19 impact on different communities and the importance of vaccine uptake. Incorporating findings from Hatmal et al. (2022) could enrich this section by providing a broader context of vaccine hesitancy and adverse effects within Arab populations, which could be relevant given the diverse communities studied.

**Methods:** The methodology is well-detailed, offering insight into the community-based survey approach. However, the selection criteria and data collection methods could be further justified. Discussing the machine learning tools used by Hatmal et al. (2022) for predicting post-vaccination adverse effects could offer a methodological comparison or improvement suggestion, especially in analyzing factors associated with vaccine initiation.

**Results:** The results section effectively presents key findings on vaccine uptake and associated factors. To enhance the discussion on vaccine hesitancy and medical mistrust, incorporating insights from J. Al-Awaida et al. (2021) regarding the impact of SARS-CoV-2 variants could provide a deeper understanding of vaccine hesitancy dynamics over time, especially considering variant-related concerns.

**Discussion:** This section thoughtfully interprets the study findings, comparing them with existing literature. Including a discussion on the findings of Hatmal et al. (2022) and J. Al-Awaida et al. (2021) could broaden the analysis. For instance, examining the role of misinformation and attitudes towards vaccination in the context of variant emergence could offer additional explanations for observed vaccine hesitancy.

**Limitations:** The manuscript acknowledges its limitations, including the sampling approach and the adaptation of survey measures. Further discussion on the limitations mentioned in Hatmal et al. (2022) and J. Al-Awaida et al. (2021), such as the predictive value of certain predisposing factors on adverse vaccine effects or the influence of variants on vaccine efficacy perceptions, could provide a more comprehensive understanding of the study's constraints.

**Conclusion:** The conclusion succinctly summarizes the study's implications. It could be strengthened by explicitly mentioning how the insights from the referenced studies could inform future vaccination efforts, particularly in addressing vaccine hesitancy and improving vaccine confidence across diverse communities.

**General Comments:**

- The manuscript provides valuable insights into COVID-19 vaccine uptake among racially and ethnically diverse communities in Colorado. It highlights important factors influencing vaccination decisions, which are crucial for designing effective public health interventions.
- Incorporating comparisons and findings from Hatmal et al. (2022) and J. Al-Awaida et al. (2021) throughout the manuscript could enhance the discussion by providing additional perspectives on vaccine hesitancy, adverse effects, and the impact of COVID-19 variants.
- Future studies could benefit from exploring the role of digital tools and machine learning in predicting vaccine uptake and addressing hesitancy, as suggested by the methodologies employed in Hatmal et al. (2022).

Overall, the research offers valuable insights into community-level vaccine uptake dynamics and contributes to the broader understanding of public health interventions' effectiveness in promoting COVID-19 vaccination. However, there are several areas where the study could be strengthened:

1. **Comparison with Existing Literature**: The paper could benefit from a more thorough comparison with existing studies, specifically in discussing its findings in the context of reported adverse effects and attitudes following COVID-19 vaccination among different populations. References such as Hatmal et al. (2022) and Al-Awaida et al. (2021) provide relevant insights into adverse effects, attitudes, and the impact of SARS-CoV-2 variants which could enhance the discussion around vaccine hesitancy and social norms.
2. **Limitations**: The study acknowledges its limitations, including potential biases due to its sampling approach and the generalizability of the findings. It is commendable that the paper discusses these limitations; however, it could further explore the impact of these limitations on the study's findings and propose strategies for addressing them in future research.
3. **Predictive Modeling**: While the paper effectively uses logistic regression to identify factors associated with vaccine initiation, incorporating predictive modeling techniques, as discussed in Hatmal et al. (2022) using machine learning tools, could provide deeper insights into the likelihood of vaccine initiation based on predisposing factors. This approach could add a novel dimension to the study by offering predictive analytics to identify individuals at risk of vaccine hesitancy.
4. **Cultural and Socio-economic Factors**: The paper could expand its discussion on the role of cultural and socio-economic factors in vaccine hesitancy and uptake. Integrating findings from Al-Awaida et al. (2021), which examines the correlates of SARS-CoV-2 variants on health outcomes across different continents, could provide a more nuanced understanding of how these factors influence vaccine attitudes and behaviors in diverse communities.

In conclusion, the paper makes a significant contribution to understanding COVID-19 vaccine uptake in racially and ethnically diverse communities. By addressing these suggested areas for improvement, future iterations of this research could offer even more comprehensive insights into effective strategies for promoting vaccine uptake and addressing public health disparities.

**references:**

1. J. Al-Awaida, W., Jawabrah Al Hourani, B., Swedan, S., Nimer, R., Alzoughool, F., J. Al-Ameer, H., ... & R. Hadi, N. (2021). Correlates of SARS-CoV-2 Variants on Deaths, Case Incidence and Case Fatality Ratio among the Continents for the Period of 1 December 2020 to 15 March 2021. *Genes*, *12*(7), 1061.
2. Hatmal, M. M. M., Al-Hatamleh, M. A., Olaimat, A. N., Mohamud, R., Fawaz, M., Kateeb, E. T., ... & Bindayna, K. M. (2022). Reported adverse effects and attitudes among Arab populations following COVID-19 vaccination: a large-scale multinational study implementing machine learning tools in predicting post-vaccination adverse effects based on predisposing factors. *Vaccines*, *10*(3), 366.
