# Supplementary material for: Factors in COVID-19 vaccine uptake in five racial/ethnic Colorado communities: A report from the Colorado CEAL project
Source: PLoS One. 2024 Jun 12;19(6):e0305160. doi: 10.1371/journal.pone.0305160 (PMC11168616; doi:10.1371/journal.pone.0305160)
Supplement: Attachment — Submitted filename: Response to Reviewers_2024-5-17.docx [file pone.0305160.s004.docx]

| **Reviewer comments** | **Authors’ Response** |
| --- | --- |
| **Editor’s Comments** |  |
| **Clarity and Structure**: The manuscript could benefit from clearer section headings and subheadings to enhance readability. The flow between sections appears disjointed, making it challenging for readers to follow the progression of the study. | We have revised existing subheadings and added several to facilitate easier readability of the discussion section, as well as other portions of the manuscript. |
| **Data Analysis**: The methods section could provide more detailed information on the statistical analysis to enhance transparency. For instance, specifying the criteria for including variables in the multivariable model could clarify the analytical choices made. | The methods for data analysis already includes the following text defining the criteria for including variables in the multivariable model: “The final multivariable model was adjusted for demographics (age, gender, education, income), community, and any predictors that were identified to be associated (p<0.2) with the outcome in bivariate analyses.” i.e., the demographic variables were decided a priori (regardless of bivariate association) and additional variables were included on the basis of having a p-value <0.2 in bivariate analyses, within an overarching goal to limit parameters according to the frequency of outcome in the less common group (e.g., non-uptake of vaccination). |
| **Sample Representativeness**: Concerns about the representativeness of the sample could be addressed more thoroughly. While the study targets specific racial/ethnic communities, it's unclear how these groups were selected and if they accurately represent the broader population's vaccine attitudes and behaviors. | Thank you for noting this concern. These groups were selected for the disparities they experienced during the COVID-19 pandemic and potential for intervention under the National Institutes of Health CEAL alliance. We have added more detail in the Participants section. ***Lines 94-100.***  We also note the limitations of these communities for representing the broader population in the limitations section. ***Lines 320-341.*** |
| **Limitations**: While some limitations are acknowledged, a more comprehensive discussion on how these limitations impact the study's findings and implications would strengthen the manuscript. For example, considering the potential biases introduced by the survey methodology or the impact of external factors on vaccine uptake during the study period. | We have greatly expanded our discussion of the limitations of our study and the implications on our findings. ***See lines 320-360.*** |
| **Implications for Practice**: The manuscript could more explicitly link its findings to practical applications. Suggestions for how healthcare providers, policymakers, and community leaders can use this information to improve vaccine uptake strategies would be valuable. | We have further expanded our discussion of the implications of this study on practice. We have noted both strategies and messengers (e.g., providers and public health) who can incorporate these findings in their efforts to increase vaccination coverage. ***See newly sub-headed “Implications for Practice”, specifically lines 361-377.*** |
| **Literature Review**: The introduction and discussion could be strengthened by incorporating a broader range of literature, especially recent studies that offer contrasting or complementary findings. This would help situate the study within the current research landscape more effectively. | Thank you for this opportunity to update our work with more recent published studies that have come out since our work has been under review. We have focused our updates primarily in the discussion as these additions are contemporaneous to our findings and in fact help to both contextualize our findings given when the study was conducted and highlight areas of concurrence and divergence around COVID-19 vaccination uptake in such underserved communities.  ***See lines 50-54, lines 280-293.*** |
|  |  |
| **Reviewer 1** |  |
| 1) The manuscript is well written.  2) The results and methodology are convincing. | Thank you for these overall positive comments on the pieces. |
| 3) The only point that is concerning about the manuscript is the small sample size that were studied; 841  participants were involved in the study but 29 participants were excluded from the study, making the  total participants 812. Hence, although the results are convincing, but I don’t believe that the 812  participants are enough to generalize the results that authors are presenting.  4) Hence my recommendation, is to involve more participants (if possible), this would enhance the  strength of the manuscript and its conclusions. | This manuscript reports on baseline data for a large community-engaged intervention study that was powered to detect clinically meaningful differences between the pre-and post-intervention periods. We are unable to involve more participants due to the dynamic context of the COVID-19 pandemic (data was collected in 2021 and the context has changed since then) and substantial survey and research burden to the community to collect data such as this. Rather, we have reviewed our discussion and conclusions to ensure we are not over-generalizing from this baseline data and noted the moderate sample size in our limitations. ***See lines 259, 290; 295-297 and 306-317.*** |
|  |  |
| **Reviewer 2** |  |
| The manuscript presents a clear and structured investigation into the motivators, concerns, and factors associated with COVID-19 vaccine initiation across diverse communities in Colorado. After reviewing the manuscript "Factors in COVID-19 Vaccine Uptake in Five Racial/Ethnic Colorado Communities: A report from the Colorado CEAL Project," we have identified several areas that warrant further attention to strengthen the manuscript: | Thank you for these constructive suggestions. |
| 1. Your manuscript provides a clear overview of the study's objectives and findings. The use of community-based data collection and logistic regression models is well-articulated. To enhance clarity, consider providing more explicit connections between the literature review and the study's specific contributions to the field. | We have added to the end of our introduction to more clearly draw this connection. ***See lines 66-70 and 79-80.*** |
| 2. The community connector model for participant recruitment is innovative but may introduce selection bias, limiting the generalizability of the findings. A discussion on how this sampling approach might have influenced the results and how future studies could address this limitation would be beneficial. | Thank you for noting this. We have added a discussion of the limitations of our sampling approach through the community connectors model for recruitment ***See lines 324-341.*** |
| 3. The application of bivariate and multivariable logistic regression models is appropriate. However, a more detailed rationale for the selection of variables included in the models, along with any assumptions or limitations of the statistical methods used, would provide greater transparency and reproducibility. | The methods for data analysis already includes the following text defining the criteria for including variables in the multivariable model: “The final multivariable model was adjusted for demographics (age, gender, education, income), community, and any predictors that were identified to be associated (p<0.2) with the outcome in bivariate analyses.” i.e., the demographic variables were decided a priori (regardless of bivariate association) and additional variables were included on the basis of having a p-value <0.2 in bivariate analyses, within an overarching goal to limit parameters according to the frequency of outcome in the less common group (e.g., non-uptake of vaccination). We did expand our reporting of the rationale (e.g., based on observed difference in early uptake of COVID-19 vaccines) and intent to limit parameters in the model. ***See lines 164-169.*** |
| 4. Expanding the literature review to include recent studies on vaccine uptake in similar and diverse contexts could provide a more comprehensive background and highlight the study's unique contributions more effectively. | We have incorporated more national data on racial and ethnic disparities in the US  ***See lines 50-54.*** |
| 5. The community-based approach and the focus on specific racial/ethnic communities in Colorado are notable strengths of the study. Elaborating on how these aspects bring new insights into vaccine uptake research could underscore the manuscript's innovativeness. | We have elaborated on the intention of this approach to enhance our sample and prepare for greater retention in a multi-wave survey in our strengths section.  ***See lines 324-341.*** |
| 6. The study's exemption by the Colorado Multiple Institutional Review Board (COMIRB) is noted. Including more details on the ethical considerations, especially in terms of participant consent and data protection, would enhance the manuscript's ethical rigor. | We have expanded our discussion of the participant consent and data protection procedures in place for this study. ***See lines 77-83.*** |
| 7. The manuscript effectively utilizes data to support its findings. Ensuring that all figures and tables are clearly labeled and directly support the narrative will further enhance the presentation and interpretation of the results. | Thank you for this feedback. We have reviewed all of our figures and labels and ensured this as suggested. |
| 8. The manuscript references relevant literature, but ensuring the inclusion of the most current and directly related studies will strengthen the background and support for the study's findings. | As noted above, we have expanded our introduction and especially the discussion section to incorporate relevant recent published literature that contextualizes our findings. |
| 9. The reliance on community connectors for recruitment, while community-engaged, may limit the representativeness of the sample. Addressing how this approach may influence the findings and suggesting ways to mitigate such limitations in future research would be valuable. | As noted above, we have added a discussion of the limitations of our sampling approach through the community connectors model for recruitment. ***See lines 324-341 in the Strengths and Limitations.*** |
| 10. The adaptation of measures from longer validated instruments is practical but may affect their reliability and validity. Discussing the implications of these adaptations on the study's conclusions would provide a more nuanced understanding of the findings. | We have expanded our discussion of the limitations and strengths of the present study, including the adaptation of these measures. ***See newly sub-headed section “Strengths and Limitations”, specifically lines 348-350.*** |
| 11. The specific context and methods of the study may limit the generalizability of the findings. A discussion on the applicability of the results to other settings and populations would provide valuable insights for readers. | We have expanded our discussion of the limitations and strengths of the present study. ***See newly sub-headed section “Strengths and Limitations”.*** |
| Addressing these points will enhance the manuscript's clarity, rigor, and contribution to the field, making it a valuable addition to the literature on COVID-19 vaccine uptake in diverse communities. | Thank you for these suggestions. We appreciate these constructive comments and the opportunity to strengthen this work. |
|  |  |
| **Reviewer 3** |  |
| Suggested references   1. J. Al-Awaida, W., Jawabrah Al Hourani, B., Swedan, S., Nimer, R., Alzoughool, F., J. Al-Ameer, H., ... & R. Hadi, N. (2021). Correlates of SARS-CoV-2 Variants on Deaths, Case Incidence and Case Fatality Ratio among the Continents for the Period of 1 December 2020 to 15 March 2021. Genes, 12(7), 1061. 2. Hatmal, M. M. M., Al-Hatamleh, M. A., Olaimat, A. N., Mohamud, R., Fawaz, M., Kateeb, E. T., ... & Bindayna, K. M. (2022). Reported adverse effects and attitudes among Arab populations following COVID-19 vaccination: a large-scale multinational study implementing machine learning tools in predicting post-vaccination adverse effects based on predisposing factors. Vaccines, 10(3), 366. | Thank you for suggesting these papers which we read with great interest and assessed for relevance to our reported study. We determined that incorporating these references would diverge significantly from the scope of the reported study and decided not include them in our manuscript. Rather, we assessed each of the following comments pointing to these references to instead address the spirit of the reviewer’s comments, such as the methodological rigor or the context in other literature. |
| Based on the detailed review of the manuscript "Factors in COVID-19 Vaccine Uptake in Five Racial/Ethnic Colorado Communities: A report from the Colorado CEAL Project," here are the comments, limitations, and suggestions for improvement. The manuscript provides valuable insights into vaccine uptake within diverse communities in Colorado, focusing on various motivators and barriers to COVID-19 vaccination. It employs a robust community-based data collection methodology, ensuring relevant and contextual data gathering from targeted communities. | Thank you for pointing out the strengths of our community-based data collection. We agree this is valuable for ensuring relevance in and representation of these communities. |
| Abstract: The abstract concisely outlines the study's purpose, methodology, results, and implications. It successfully highlights the importance of tailored community messaging to improve vaccination rates. However, it could benefit from mentioning the study's limitations to provide a more balanced overview. | We agree and have briefly acknowledged our moderate sample size as a limitation in the conclusion of the abstract. ***See line 14.*** |
| Introduction: The introduction effectively sets the stage for the study by discussing the COVID-19 impact on different communities and the importance of vaccine uptake. Incorporating findings from Hatmal et al. (2022) could enrich this section by providing a broader context of vaccine hesitancy and adverse effects within Arab populations, which could be relevant given the diverse communities studied. | Thank you for noting the strengths of our introduction. |
| Methods: The methodology is well-detailed, offering insight into the community-based survey approach. However, the selection criteria and data collection methods could be further justified. Discussing the machine learning tools used by Hatmal et al. (2022) for predicting post-vaccination adverse effects could offer a methodological comparison or improvement suggestion, especially in analyzing factors associated with vaccine initiation. | We have revised to add clarity to our participant eligibility criteria, recruitment/selection procedures, and data collection. ***See lines 95-118.*** |
| Results: The results section effectively presents key findings on vaccine uptake and associated factors. To enhance the discussion on vaccine hesitancy and medical mistrust, incorporating insights from J. Al-Awaida et al. (2021) regarding the impact of SARS-CoV-2 variants could provide a deeper understanding of vaccine hesitancy dynamics over time, especially considering variant-related concerns. | Thank you for noting our effective presentation of our key findings. |
| Discussion: This section thoughtfully interprets the study findings, comparing them with existing literature. Including a discussion on the findings of Hatmal et al. (2022) and J. Al-Awaida et al. (2021) could broaden the analysis. For instance, examining the role of misinformation and attitudes towards vaccination in the context of variant emergence could offer additional explanations for observed vaccine hesitancy. | We appreciate the reviewer’s comment on our discussion section. We have also added additional comparisons from recent literature to further contextual our findings in the current science. |
| Limitations: The manuscript acknowledges its limitations, including the sampling approach and the adaptation of survey measures. Further discussion on the limitations mentioned in Hatmal et al. (2022) and J. Al-Awaida et al. (2021), such as the predictive value of certain predisposing factors on adverse vaccine effects or the influence of variants on vaccine efficacy perceptions, could provide a more comprehensive understanding of the study's constraints. | We have expanded our discussion of the limitations and strengths of the present study. ***See newly sub-headed section “Strengths and Limitations”.*** |
| Conclusion: The conclusion succinctly summarizes the study's implications. It could be strengthened by explicitly mentioning how the insights from the referenced studies could inform future vaccination efforts, particularly in addressing vaccine hesitancy and improving vaccine confidence across diverse communities. | We have more clearly stated how these findings will inform community co-created messaging to promote vaccination uptake in these communities. ***See lines 380-386*** |
| General Comments: |  |
| - The manuscript provides valuable insights into COVID-19 vaccine uptake among racially and ethnically diverse communities in Colorado. It highlights important factors influencing vaccination decisions, which are crucial for designing effective public health interventions. | Thank you. |
| - Incorporating comparisons and findings from Hatmal et al. (2022) and J. Al-Awaida et al. (2021) throughout the manuscript could enhance the discussion by providing additional perspectives on vaccine hesitancy, adverse effects, and the impact of COVID-19 variants. | While we agree that these papers have provided important perspectives on adverse events and COVID-19 variants in other context, we reiterate that these are not appropriate for the present study on factors in vaccination in underserved communities in Colorado. |
| - Future studies could benefit from exploring the role of digital tools and machine learning in predicting vaccine uptake and addressing hesitancy, as suggested by the methodologies employed in Hatmal et al. (2022). | We agree that digital tools and machine learning have an important place in vaccine hesitancy surveillance and tracking of adverse events and hopeful they will be used more frequently in future studies. However, these are outside the scope of the present study. |
| Overall, the research offers valuable insights into community-level vaccine uptake dynamics and contributes to the broader understanding of public health interventions' effectiveness in promoting COVID-19 vaccination. However, there are several areas where the study could be strengthened: |  |
| 1. Comparison with Existing Literature: The paper could benefit from a more thorough comparison with existing studies, specifically in discussing its findings in the context of reported adverse effects and attitudes following COVID-19 vaccination among different populations. References such as Hatmal et al. (2022) and Al-Awaida et al. (2021) provide relevant insights into adverse effects, attitudes, and the impact of SARS-CoV-2 variants which could enhance the discussion around vaccine hesitancy and social norms. | As we have stated, these studies are outside the scope of our current study. |
| 2. Limitations: The study acknowledges its limitations, including potential biases due to its sampling approach and the generalizability of the findings. It is commendable that the paper discusses these limitations; however, it could further explore the impact of these limitations on the study's findings and propose strategies for addressing them in future research. | Thank you. We have expanded our limitations and strengths section. ***See lines 320-360.*** |
| 3. Predictive Modeling: While the paper effectively uses logistic regression to identify factors associated with vaccine initiation, incorporating predictive modeling techniques, as discussed in Hatmal et al. (2022) using machine learning tools, could provide deeper insights into the likelihood of vaccine initiation based on predisposing factors. This approach could add a novel dimension to the study by offering predictive analytics to identify individuals at risk of vaccine hesitancy. | Thank you for this suggestion. While this is outside the scope of the current study, we will consider these methodologies for future studies in this space. |
| 4. Cultural and Socio-economic Factors: The paper could expand its discussion on the role of cultural and socio-economic factors in vaccine hesitancy and uptake. Integrating findings from Al-Awaida et al. (2021), which examines the correlates of SARS-CoV-2 variants on health outcomes across different continents, could provide a more nuanced understanding of how these factors influence vaccine attitudes and behaviors in diverse communities. | Thank you for this suggestion. While this is outside the scope of the current study, we will consider these methodologies for future studies in this space. |
| In conclusion, the paper makes a significant contribution to understanding COVID-19 vaccine uptake in racially and ethnically diverse communities. By addressing these suggested areas for improvement, future iterations of this research could offer even more comprehensive insights into effective strategies for promoting vaccine uptake and addressing public health disparities. | Thank you. |
